# *Mycobacterium tuberculosis* Contaminant Risk on Bone Marrow Aspiration Material from Iliac Bone Patients with Active Tuberculous Spondylitis

**DOI:** 10.1155/2016/3852940

**Published:** 2016-05-11

**Authors:** Ahmad Jabir Rahyussalim, Tri Kurniawati, Andriansjah Rukmana

**Affiliations:** ^1^Department of Orthopaedic and Traumatology, Faculty of Medicine, University of Indonesia, Cipto Mangunkusumo Hospital, Jakarta 10320, Indonesia; ^2^Stem Cell Integrated Medical Service Unit, Cipto Mangunkusumo Hospital, Faculty of Medicine, Universitas Indonesia, Jakarta 0320, Indonesia; ^3^Department of Clinical Microbiology, Faculty of Medicine, University of Indonesia, Jakarta 10320, Indonesia

## Abstract

There was a concern on* Mycobacterium tuberculosis* spreading to the bone marrow, when it was applied on tuberculous spine infection. This research aimed to study the probability of using autologous bone marrow as a source of mesenchymal stem cell for patients with tuberculous spondylitis. As many as nine patients with tuberculous spondylitis were used as samples. During the procedure, the vertebral lesion material and iliac bone marrow aspirates were obtained for acid fast staining, bacteria culture, and PCR (polymerase chain reaction) tests for* Mycobacterium tuberculosis* at the Clinical Microbiology Laboratory of Faculty of Medicine Universitas Indonesia. This research showed that there was a relationship between diagnostic confirmation of tuberculous spondylitis based on the PCR test and bacterial culture on the solid vertebral lesion material with the PCR test and bacterial culture from the bone marrow aspirates. If the diagnostic confirmation concluded positive results, then there was a higher probability that there would be a positive result for the bone marrow aspirates, so that it was not recommended to use autologous bone marrow as a source of mesenchymal stem cell for patients with tuberculous spondylitis unless the PCR and culture examination of the bone marrow showed a negative result.

## 1. Background

Invasion of* Mycobacterium tuberculosis* bacteria to vertebral body (tuberculous spondylitis) was one of the causes of vertebral deformities that decreased the quality of life of the patient [1–3]. The Subroto Sapardan Total Treatment for tuberculous spondylitis cases with severe defects used the tricortical bone autograft that was obtained from the iliac bone in order to fill the gap defect [4], although there were still around 5% of fusion failure cases after treatment. Surgical intervention was done by indication such as paravertebral abscess, neurological problem, and unstable and spine deformity.

Most of the estimated number of cases of TB occurred in Asia (55%) and Africa (30%), and smaller proportions were registered in Eastern Mediterranean Region (7%), European Region (4%), and Region of the Americas (3%.). Indonesia was one of the big countries which had tuberculous infection.

Mesenchymal stem cell was a special cell that was produced from the bone marrow, fat tissue, umbilical blood, amniotic fluid, placenta, teeth pulp, tendon, synovial membrane, and muscle [5]. The bone marrow was a source of mesenchymal stem cell that has been widely used for tissue reconstruction for bone defect healing due to its composition of osteogenic precursor cells [5, 6]. Bone marrow was obtained easily by aspiration technique so that traumatic procedures could be avoided. After being aspirated, the bone marrow would be cultured in vitro so that the cell quantity increased, hence an ideal source for autologous bone tissue reconstruction [7, 8].

Several studies stated that the bone tissue formed after implantation of the mesenchymal stem cells originated from bone marrow stromal cells possesses osteogenic capacity similar to the autologous bone graft [8]. This research showed that the bone marrow stromal cells proved to be efficient in increasing the bone healing ability after it was planted to where the bone is broken [8–10].

The success of using the autologous mesenchymal stem cell originated from the bone marrow for fusion purposes in degenerative disease was the stem of this research. However, mesenchymal stem cell required the usage of bone marrow from the patients themselves (autologous), whereas the laboratory used for culture necessitates an infection-clear bone marrow. Concerns on* Mycobacterium tuberculosis* infection spreading to the bone marrow lead to the need of studying the utilization of autologous bone marrow as a source of mesenchymal stem cell.

This research aimed to prove the existence of* Mycobacterium tuberculosis* in the bone marrow of patients with tuberculous spondylitis and study the application of autologous bone marrow as a source of mesenchymal stem cell on patients with tuberculous spondylitis.

## 2. Materials and Methods

This was a descriptive-observational study that was done at Cipto Mangunkusumo Hospital, Jakarta, from January to October 2014. The samples were lesions and bone marrow materials from patients with tuberculous spondylitis that met the inclusive criteria, which were patients with tuberculous spondylitis, having surgery indications, never having been in surgery before on any locations because of suspected tuberculous spondylitis, aged between 10 and 60 years, and having signed the informed consent. Surgery procedure was included into criteria because of need to collect sample for lesion and bone marrow blood. The number of samples was counted using proportional difference (80%), which were 18 unit samples from 9 patients.

Determination of study participants was done consecutively in outpatient clinic. The unit study samples such as debris material, abscess, and granulation tissue and bone marrow aspirations were taken on the same day of the surgery.

**Figure 1:**
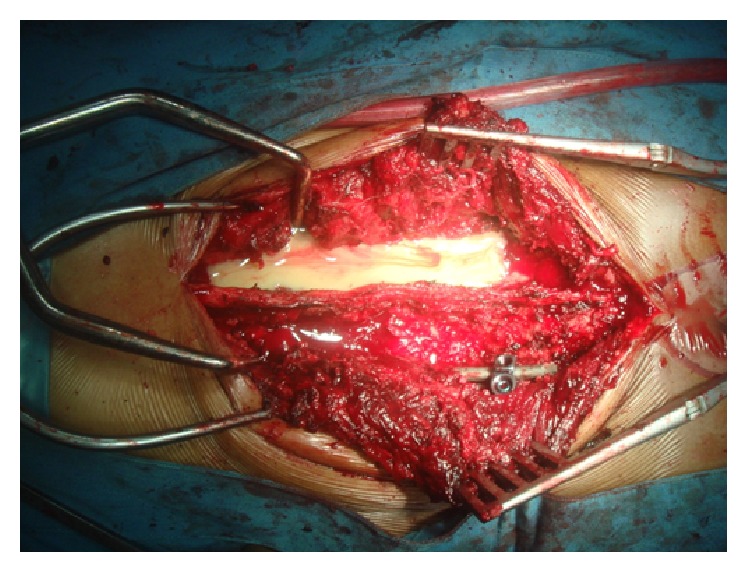
Tuberculous spondylitis lesion. There was about 50 cc pus as liquid lesion. Solid lesion does not appear in the picture.

Some material from vertebral lesion was collected that consists of solid and liquid material. Solid material consisted of sequestered and necrotic bone. Liquid material consisted of blood, pus, and lysis material from soft tissue (Figure 1). The lesion material (debris, abscess, and granulation tissue) was obtained invasively during surgery procedure, and the bone marrow sample was done with minimal invasive procedure using the trocar on the iliac crest region.

**Figure 2:**
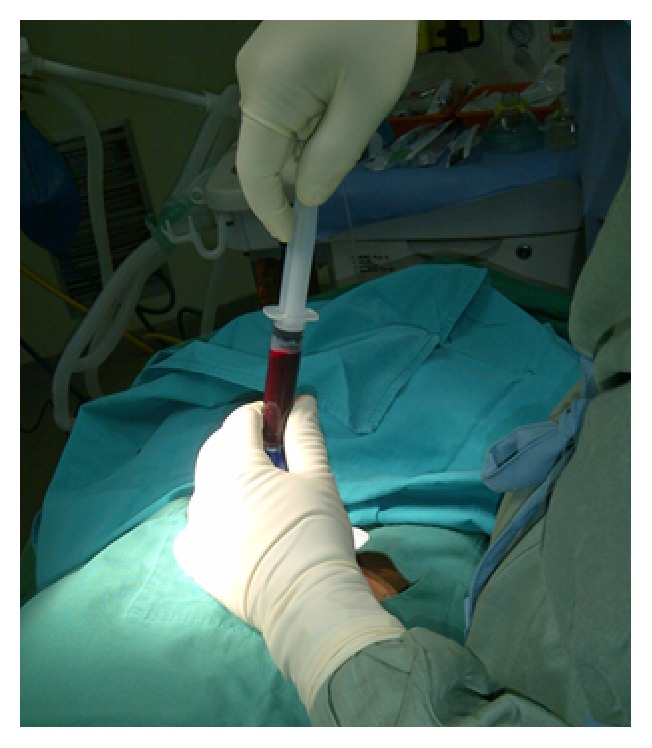
Procedure of iliac bone marrow aspiration on patient with tuberculous spondylitis. It shows that 10 cc bone marrow blood was harvested.

The three steps of bone marrow aspiration procedure (Figure 2) were developed as follows: (1) trocar with 84 mm size was inserted into iliac crest bone at the point of posterior superior iliac crest (PSIS), (2) as much as 10 mL of bone marrow blood was aspirated by trocar using vacuum maneuver into sterile syringe, and (3) the inside of syringe was rinsed by anticoagulants. The sample collection was done while patient was in general anesthesia. Samples (lesion and bone marrow blood) were then sent to the Clinical Microbiology Laboratory for acid fast staining, culture examination, and PCR for* Mycobacterium tuberculosis* [11, 12]. PCR amplification for IS6110 gene was performed by set of primers TB1,5′-CTCGCGAGCGTAGGCGTCGG-3′ and TB2,5′-CTCGTCCAGCGCCGCTTCGG-3′, which amplify a fragment of 130-base pair (bp) of the target gene.

The laboratory was accredited for culture and 1st and 2nd line antituberculosis drug resistance examination by Supranational Referral Laboratory (SRL) IMVS, Adelaide, Australia. It was accredited as a laboratory that was recommended by WHO to do certification for tuberculosis laboratory. The diagnosis of tuberculous spondylitis was upheld by culture examination based on international standard for tuberculosis care (ISTC).

If the acid fast staining, culture examination, or the PCR examination for the* Mycobacterium tuberculosis* was negative, then the sample would not be used as a sample, and if only just one of them was positive then the sample will be used as a research sample.

## 3. Result

The participants of this study were 5 females and 4 males. The youngest participant was 21 years old and the oldest was 38 years old. Most of cases were infected at thoracolumbar area. There was no sign of infection at iliac region. Generally participants have history of tuberculous lung which was not treated well. Antituberculous drug was not taken according to the recommended guidelines.

According to the examination result on the solid lesion material from the 9 cases of tuberculous spondylitis (Table 1), there are 9 (9) positive histopathology tests, 1 (9) positive culture test, 2 (9) positive PCR tests, and no solid material positive according to acid fast staining (AFS) test.

According to the result of the liquid lesion material from the 9 cases of tuberculous spondylitis, there are no liquid material positive results according to culture examination, 3 (9) positive PCR tests, and no (9) positive AFS test.

According to the bone marrow sample from the 9 bone marrow blood aspirations of tuberculous spondylitis iliac bone (Table 2), there is 1 (9) positive culture test, 1 positive PCR test of 9 bone marrow blood aspirations, and none after AFS.

## 4. Discussion

The diagnosis of tuberculous spondylitis in this research was confirmed by histopathologic examination [13, 14]; thus it can be concluded that 7 from 9 samples (77,78%) are nonactive spondylitis, whiles 2 out of 9 samples (22,22%) are active spondylitis confirmed by positive* Mycobacterium tuberculosis* culture [13, 15].

From the bone marrow examination it can be seen that there is a relation between the positive PCR and culture result for solid lesion with the PCR and culture result from the bone. This means that PCR and/or culture of solid lesion might be the marker for the bone marrow or, on the other hand, it means that if we found a positive result from PCR and/or culture from the solid lesion, then it is highly probable that we get a similar result from the PCR and/or culture from the bone marrow.

From 9 cases of tuberculous spondylitis, according to the histopathology examination, there is 1 test that gives positive result from the bone marrow. This means that there is a migration of the* Mycobacterium tuberculosis* from the lesion to the bone marrow. The migration can be caused by some conditions such as immunocompromised general condition, blood flow turbulence, and inadequate antibiotic regime.

### 4.1. Immunocompromised Condition

Being immunocompromised is a condition where the immune response of the patient is decreased (weak) because of the consumption of immunosuppressant drugs, radiation, malnutrition, and other conditions such as cancer infections [16]. In this condition, patients experience severe cellular immunity disruption and, thus, become vulnerable to infections, including bacterial infections [16, 17]. The immunocompromised condition of cases with positive result in the lesion and bone marrow is probably caused by malnutrition so that the* Mycobacterium tuberculosis* infection spreads to other regions, especially to the bone marrow, instead of being contained inside the original lesion.

Application of MSCs on tuberculosis positive bone defect showed that spondylitis tuberculosis supports enhanced ossification and new bone formation by stimulating calcium metabolism in lesions. MSCs hold an immunoregulatory capacity and elicit immunosuppressive effects in a number of situations. There was no evidence of tuberculosis reactivation after implantation of MSCs on infection vertebral bone defect [18].

In immunocompromised individuals, the immunomodulatory activities of MSCs have raised safety concerns regarding the greater risk of primary viral infection and viral reactivation, which is a major cause of mortality after allogeneic transplantation [19, 20].

### 4.2. Blood Flow Turbulence Theory

The existence of the* Mycobacterium tuberculosis* on blood flow will cause the bacteria spread systemically. The folding inside the blood vessel anatomically can become microenvironment that support and cause the bacteria to be trapped in the vessel and, if continued, can induce bacterial proliferation in the blood, causing sepsis, and ultimately infect the bone marrow [21].

### 4.3. Inadequate Antibiotic Regime

The existence of the* Mycobacterium tuberculosis* in the organs depends on the dosage and duration of antituberculosis drugs (ATD) given [13, 16]. All of the patients in this research were given ATD for at least 2 weeks before being included in the procedure, which is probably why the* Mycobacterium tuberculosis* keep growing and spread to the bone marrow. This means that the ATD that were given were not effective yet in killing* Mycobacterium tuberculosis* or the bacterium itself may have resistance ability towards the ATD, hence causing the ATD to not work effectively.

In this research it is not proven that there is a difference in the potency of the culture of the bone marrow result from the iliac bone that came from the patients with tuberculous spondylitis compared to patients with normal condition; instead, this research proves the existence of the* Mycobacterium tuberculosis* in the bone marrow that came from the patient with tuberculous spondylitis after positive results of culture and PCR test, thus knowing that the contamination of the bacteria within the bone marrow is important before processing the bone marrow as a source of autologous mesenchymal stem cell so that it will not cause contamination to the cell that will be cultured, as well as the operator and the laboratory facilities used.

### 4.4. Isolation Methods

The processing and expansion of the cells will take place at the Good Manufacturing Practice (GMP) Facility of the Stem Cell Medical Technology Integrated Service Unit, Cipto Mangunkusumo Hospital. Final product release is based on the following criteria: surface marker expression (>90% of cells CD73+/CD90+/CD105+, ≤1% of cells CD45+, and ≤0.01% of cells CD3+), absence of microbial contamination using culture and mycoplasma PCR, spindle shaped morphology, a colorless cell suspension devoid of cell aggregates, and no genetic abnormalities using karyotype analysis and viability [22, 23].

### 4.5. MSCs Differentiation

The standard tissue engineering approach to provide solutions for impaired fracture healing, bone restoration, and regeneration includes the utilization of growth factors, scaffolds, and mesenchymal stem cells (triangular concept). However, although the mechanical environment is discussed and is considered an important element in bone regeneration, its importance is often underestimated and it is not always given the necessary attention. Lucarelli et al. in their study showed that combination of mesenchymal stem cells and platelet-rich plasm with scaffold generated the new bone and occupies the 3 cm bone defect [24].

## 5. Conclusion

There is a potency of spreading of* Mycobacterium tuberculosis* into the bone marrow of iliac bone as much as 20 percent on tuberculous spondylitis case, so that the usage of autologous mesenchymal stem cell on tuberculous spondylitis cases needs to be well considered and decontaminated for the iliac bone marrow preparation.

## 6. Recommendation

Allogeneic stem cell should be used in patients with tuberculous spondylitis with more than one defect in the vertebral body from donors that have met the preliminary screening test. More research should be done to prove the different potency of culture result of iliac bone marrow that came from the tuberculous spondylitis patients with the normal patient.

## Figures and Tables

**Table 1:** Microbiology and histopathology result.

Sample code	Solid lesion				Liquid lesion		Bone marrow		
	AFS	PCR	Culture	HP	AFS	PCR	AFS	PCR	Culture
SU 01	−	−	−	+	−	−	−	−	−
SU 02	−	−	−	+	−	−	−	−	−
SU 03	−	+	−	+	−	−	−	+	−
SU 04	−	−	−	+	−	−	−	−	−
SU 05	−	−	−	+	−	+	−	−	−
SU 06	−	+	+	+	−	−	−	−	+
SU 07	−	−	−	+	−	−	−	−	−
SU 08	−	−	−	+	−	+	−	−	−
SU 09	−	−	−	+	−	+	−	−	−

AFS: acid fast staining; PCR: polymerase chain reaction; HP: histopathology.

**Table 2:** Proportion of solid lesion, liquid lesion, and bone marrow aspirates positive examination results.

Examination	Solid lesion	Liquid lesion	Bone marrow
AFS	0/9	0/9	0/9
Culture	1/9	none	1/9
PCR	2/9	3/9	1/9
Histopathology	9/9	none	none

AFS: acid fast staining; PCR: polymerase chain reaction; HP: histopathology.
